# Genome Mining Reveals Two Missing CrtP and AldH Enzymes in the C30 Carotenoid Biosynthesis Pathway in *Planococcus faecalis* AJ003^T^

**DOI:** 10.3390/molecules25245892

**Published:** 2020-12-12

**Authors:** Jun Ho Lee, Jin Won Kim, Pyung Cheon Lee

**Affiliations:** Department of Molecular Science and Technology, Ajou University, World Cup-ro, Yeongtong-gu, Suwon-si 16499, Korea; murolove@ajou.ac.kr (J.H.L.); no7korea@ajou.ac.kr (J.W.K.)

**Keywords:** genome mining, *Planococcus faecalis*, carotenoids

## Abstract

*Planococcus faecalis* AJ003^T^ produces glycosyl-4,4′-diaponeurosporen-4′-ol-4-oic acid as its main carotenoid. Five carotenoid pathway genes were presumed to be present in the genome of *P. faecalis* AJ003^T^; however, 4,4-diaponeurosporene oxidase (CrtP) was non-functional, and a gene encoding aldehyde dehydrogenase (AldH) was not identified. In the present study, a genome mining approach identified two missing enzymes, CrtP2 and AldH2454, in the glycosyl-4,4′-diaponeurosporen-4′-ol-4-oic acid biosynthetic pathway. Moreover, CrtP2 and AldH enzymes were functional in heterologous *Escherichia coli* and generated two carotenoid aldehydes (4,4′-diapolycopene-dial and 4,4′-diaponeurosporene-4-al) and two carotenoid carboxylic acids (4,4′-diaponeurosporenoic acid and 4,4′-diapolycopenoic acid). Furthermore, the genes encoding CrtP2 and AldH2454 were located at a distance the carotenoid gene cluster of *P. faecalis*.

## 1. Introduction

Carotenoids are the most common isoprenoid pigments, comprising colors ranging from yellow through bright orange to red [1,2]. They act as precursors for several hormones and reveal diverse biological functions in nature, such as light-harvesting, photoprotection, and coloration [3]. Biotechnologically, carotenoids have been mainly utilized as food colorants, antioxidants, and animal feed supplements [4]. Their applications in biotechnology are extended to nutraceuticals, cosmetics, and pharmaceuticals. Compared with C40 carotenoids, which comprise a backbone of 40 carbons, including lycopene and β-carotene, C30 carotenoids are rare in nature, and their biological activities, biosynthetic pathways, and regulation of gene expression remain unclear [5]. A recent study on the biological functions of C30 carotenoids, including stem cell proliferation and antioxidative activities, has attracted interest toward C30 carotenoids [6].

**Figure 1:**
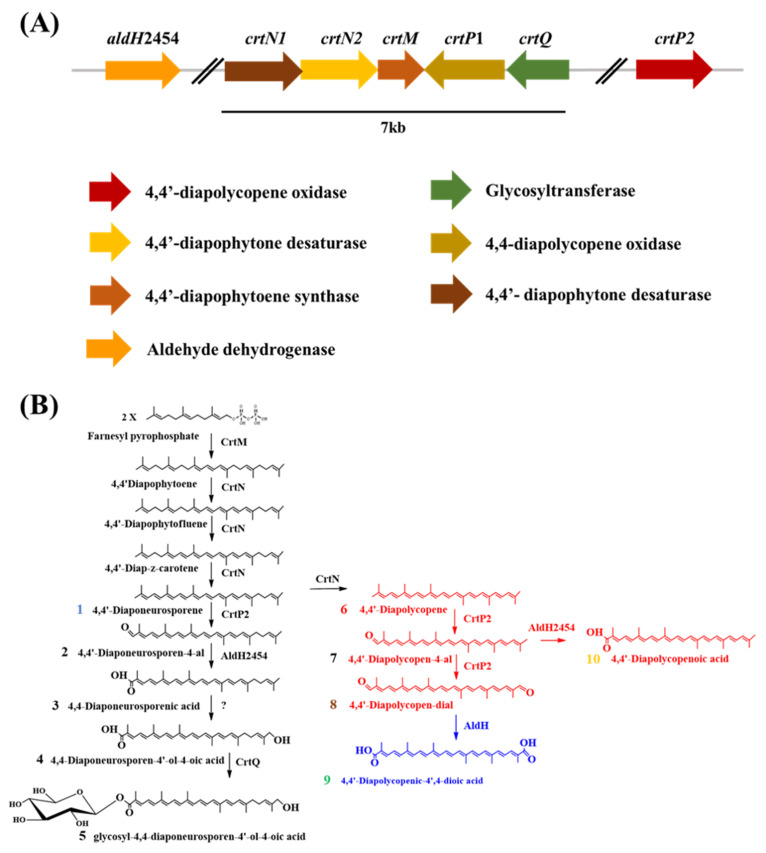
Proposed biosynthesis gene clusters and pathway of glycosyl-4,4′-diaponeurosporen-4′-ol-4-oic acid. (**A**) A whole gene cluster comprising five carotenoid pathway genes was presented with translational direction of a gene. (**B**) The biosynthetic pathway of glycosyl-4,4′-diaponeurosporen-4′-ol-4-oic acid in *P. faecalis* (in black) and the extended carotenoid pathway with CrtP2 and AldH2454 enzymes of *P. faecalis* (red) and with AldH enzyme of *S. aureus* (blue) in heterologous *E. coli*. The question mark indicates a possibility of a missing enzyme or a spontaneous step.

We recently isolated and characterized an orange bacterium, *Planococcus faecalis* AJ003^T^ [6], which produces the rare C30 carotenoid glycosyl-4,4′-diaponeurosporen-4′-ol-4-oic acid. Computational analysis of the complete genome sequence of *P. faecalis* [7] revealed a gene cluster encoding the glycosyl-4,4′-diaponeurosporen-4′-ol-4-oic acid biosynthetic pathway in the genome (Figure 1A). Biosynthesis of glycosyl-4,4′-diaponeurosporen-4′-ol-4-oic acid requires at least five enzymes (Figure 1B): 4,4′-diapophytoene synthase (CrtM), 4,4′-diapophytoene desaturase (CrtN), 4,4′-diaponeurosporene oxidase (CrtP), aldehyde dehydrogenase (AldH), and glycosyltransferase (CrtQ); however, no gene was found encoding the AldH-like enzyme in the carotenoid pathway gene cluster. Furthermore, the CrtP enzyme encoded by the *crtP* gene in the biosynthesis gene cluster was not active, unlike other pathway enzymes in heterologous *Escherichia coli* [7]. Therefore, the missing carotenoid pathway genes, which may be remotely located from the gene cluster, need to be mined.

Herein, we report that the genome mining approach successfully identified two missing genes, *crtP2* and *aldH2454*, encoding CrtP and AldH, respectively, in the glycosyl-4,4′-diaponeurosporen-4′-ol-4-oic acid biosynthetic pathway of *P. faecalis*. Complementation-based functional studies of CrtP2 and AldH enzymes were performed in heterologous *E. coli*. 

## 2. Results and Discussion

### 2.1. Identification of crtP Encoding 4,4-diaponeurosporene Oxidase 

**Figure 2:**
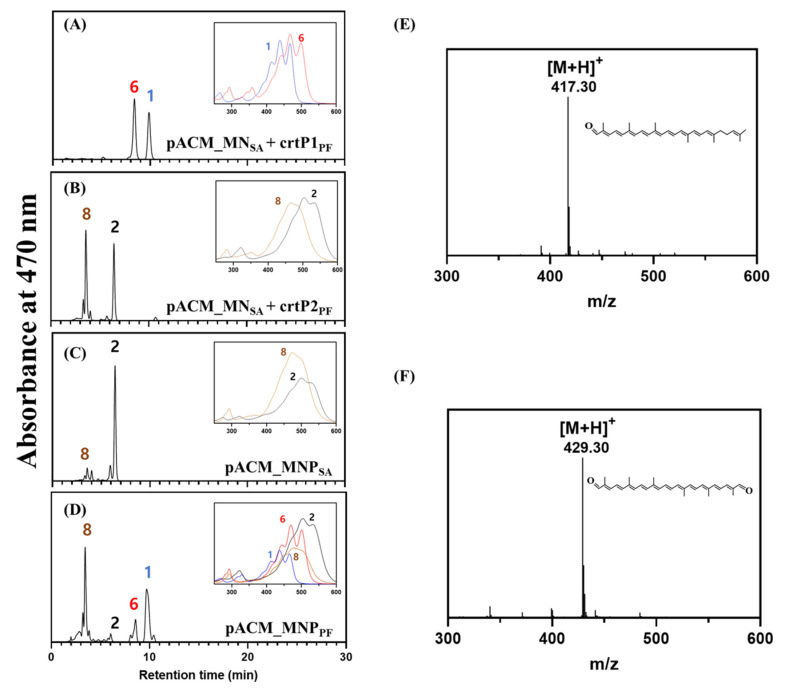
Functional analysis of CrtP2 enzyme in heterologous *E. coli* and LC-MS analysis. HPLC analysis of cell extracts of recombinant *E. coli* expressing (**A**) crtM_SA_-crtN_SA_ + crtP1_PF_, (**B**) crtM_SA_-crtN_SA_ + crtP2_PF,_ (**C**) crtM_SA_-crtN_SA_-crtP_SA_ of *S. aureus* (as a positive control), and (**D**) crtM_PF_-crtN_PF_-crtP_PF_. The insert UV/VIS spectra in the panels (**A**–**D**) correspond to peaks in the HPLC chromatogram. The peak numbers (1, 2, 6 and 8) refer to the corresponding structures in Figure 1B. LC-MS analysis of (**E**) 4,4′-diaponeurosporene-4-al (2; [M + H]^+^ = 417.30) and (**F**) 4,4′-diapolycopene-dial (8; [M + H]^+^ = 429.30).

Our previous study [7] revealed that, although the *crtP* gene encoding 4,4-diaponeurosporene oxidase was present in the glycosyl-4,4′-diaponeurosporen-4′-ol-4-oic acid biosynthetic gene cluster (Figure 1A), CrtP1 enzyme was not active, unlike the other three enzymes (CrtM, CrtN1, and CrtN2), in the heterologous *E. coli* expressing both crtM and crtN of *P. faecalis* as illustrated in Figure 2A. Therefore, we assumed the presence of other active *crtP* genes located remotely from the carotenoid gene cluster. To identify the active *crtP* genes, based on amino acid similarity score with the CrtP enzyme of *S. aureus* [8], putative CrtP-like enzymes were computationally explored against the genome of *P. faecalis*. One putative *crtP*-like gene was identified and named *crtP2*, to distinguish it from the inactive *crtP* gene (renamed as *crtP1*) present in the carotenoid gene cluster. 

To verify the functionality of *crtP2*, the gene was cloned into a high copy number pUCM expression vector, generating pUCM_crtP2_PF_ and was expressed in the 4,4′-diaponeurosporene (**1**)/4,4′-diapolycopene (**6**) producing *E. coli* [pACM_crtM_SA_-crtN_SA_]. As illustrated in Figure 2B, two main peaks were detected in the high performance liquid chromatography (HPLC) chromatogram of the cell extract, which had similar retention times to those of the two peaks from the control *E. coli* strain [pACM_crtM_SA_-crtN_SA_-crtP_SA_] (Figure 2C). Further analysis using UV/VIS spectroscopy and LC/MS (Figure 2E,F) confirmed that peak 2 corresponded to 4,4′-diaponeurosporene-4-al (**2**; [M + H]^+^ = 417.30) and peak 8 to 4,4′-diapolycopene-dial (**8**; [M + H]^+^ = 429.30). Peak 8 is a carotenoid pathway intermediate in the biosynthesis of glycosyl-4,4′-diaponeurosporen-4′-ol-4-oic acid (**5**) in *P. faecalis* (Figure 1B), but peak 2 is not. Several studies have reported that recombinantly expressed carotenoid pathway enzymes exhibit broad substrate specificities, in contrast to those endogenous to the hosts [7,8,9,10,11,12,13], which might be the case for the CrtP2 enzyme of *P. faecalis* in heterologous *E. coli*. Therefore, detection of 4,4′-diapolycopene-dial (**8**) and 4,4′-diaponeurosporene-4-al (**2**) in *E. coli* strongly indicates that *crtP2* is the first missing gene involved in the oxidation of 4,4′-diaponeurosporene (**1**) into 4,4′-diaponeurosporene-al (**2**) (Figure 1B) during the biosynthesis of glycosyl-4,4′-diaponeurosporen-4′-ol-4-oic acid (**5**) in *P. faecalis*. Notably, formation of the dialdehyde carotenoid 4,4′-diapolycopene-dial (**8**) (Figure 2F) indicates that the CrtP2 enzyme could introduce the second aldehyde group into a carotenoid monoaldehyde. Moreover, *crtP2* was expressed with crtM_PF_-crtN_PF_ of *P. faecalis* as a low copy number plasmid (pACM_crtM_PF_-crtN_PF_-crtP2_PF_) in *E. coli* to investigate the effect of expression level of three enzymes on the resulting carotenoid profile: unlike the two-plasmid system utilizing a high and low copy number plasmid, 4,4′-diapolycopene-dial (**8**) was the dominant carotenoid, with small amounts of 4,4′-diaponeurosporene-4-al (**2**), 4,4′-diaponeurosporene (**1**), and 4,4′-diapolycopene (**6**) (Figure 2D), thereby suggesting that balanced expression of pathway enzymes could influence the carotenoid profile. Eventually, although the CrtP enzyme has dual functions of oxygenase and AldH [14], carotenoid carboxylic acid intermediates such as 4,4′-diaponeurosporenoic acid (**3**) or 4,4′-diapolycopenoic acid (**10**) were not detected in either plasmid system. This suggests that CrtP2 may only exhibit oxidase activity while adding the aldehyde groups into 4,4′-diaponeurosporene (**2**) and 4,4′-diapolycopene (**6**), similar to CrtP from *S. aureus*.

### 2.2. Identification of aldH Encoding Aldehyde Dehydrogenase

As CrtP2 exhibited only oxygenase activity by adding aldehyde groups, other gene(s) encoding AldH enzymes that catalyze the oxidation reaction of the aldehyde group of 4,4′-diaponeurosporene-4-al (**2**) should be present in the genome of *P. faecalis*. It has been reported that *aldH* genes encoding carotenoid aldehyde dehydrogenase are remotely located from the corresponding carotenoid pathway gene clusters in *S. aureus* and *Methylomonas* sp. [8,15]. Therefore, using an approach similar to the computational identification of CrtP2, putative AldH-like enzymes were explored against the genome of *P. faecalis* with the amino acid sequence of the AldH enzyme of *S. aureus*. Genome mining, based on the high amino acid sequence similarity scores, identified four putative aldH genes, namely *aldH420*, *aldH905*, *aldH1759*, and *aldH2454*, on the genome of *P. faecalis*.

**Figure 3:**
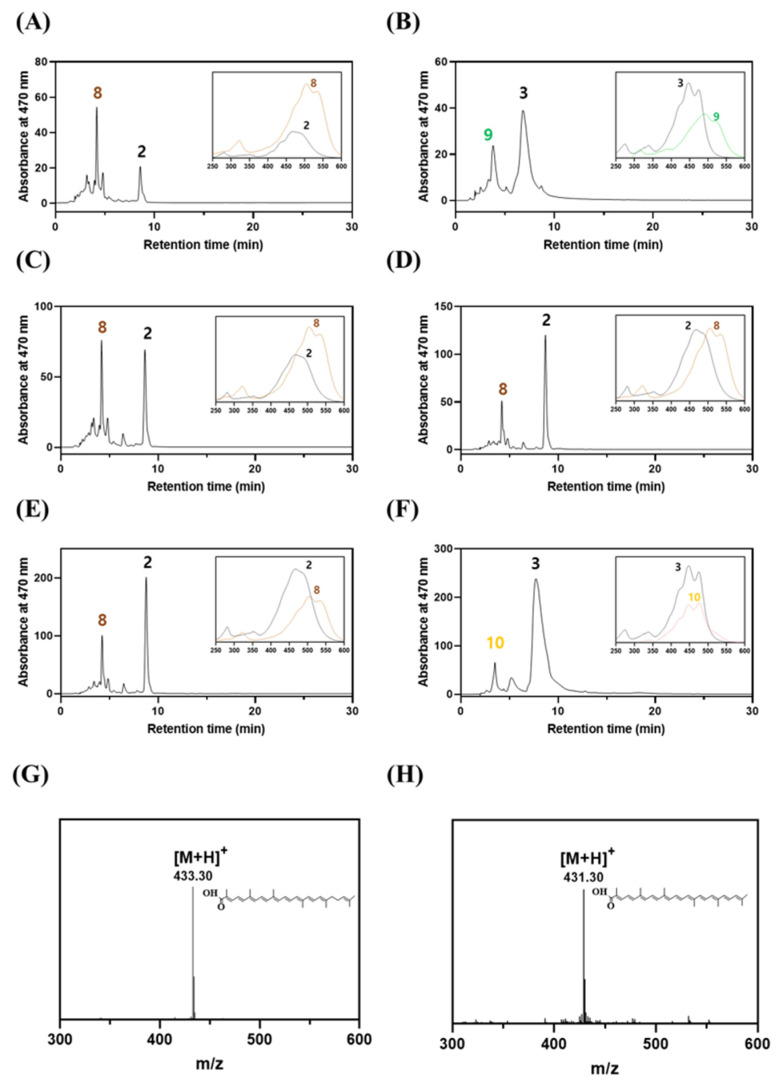
Functional analysis of four putative AldH enzymes in heterologous *E. coli* and LC-MS analysis. HPLC analysis of cell extracts of recombinant *E. coli* expressing (**A**) crtM_SA_-crtN_SA_-crtP_SA_ (as a negative control), (**B**) crtM_SA_-crtN_SA_-crtP_SA_ + aldH_SA_ of *S. aureus* (as a positive control), (**C**) crtM_SA_-crtN_SA_-crtP_SA +_ aldH420_PF_, (**D**) crtM_SA_-crtN_SA_-crtP_SA_ + aldH905_PF_, (**E**) crtM_SA_-crtN_SA_-crtP_SA_ + aldH1759_PF_, and (**F**) crtM_SA_-crtN_SA_-crtP_SA_ + aldH2454_PF_. The insert UV/VIS spectra in the panels correspond to peaks in the HPLC chromatogram. The peak numbers (2,3,8,9 and 10) refer to the corresponding structures in Figure 1B. LC-MS analysis of (**G**) 4,4′-diaponeurosporenoic acid (3; M + H]^+^ = 433.30) and (**H**) 4,4′-diapolycopenoic acid (10; [M + H]^+^ = 431.30).

To investigate the functionality of the four putative *aldH* genes, each gene was cloned into a pUCM expression vector, generating pUCM_aldH420_PF_, pUCM_aldH905_PF_, pUCM_aldH1759_PF_, and pUCM_aldH2454_PF_. The four expression vectors were then individually expressed in 4,4′-diapolycopene-dial (**8**) and 4,4′-diaponeurosporene-4-al (**2**) producing *E. coli* [pACM_crtM_SA_-crtN_SA_-crtP_SA_]. HPLC analysis revealed that only the cell extract from *E. coli* expressing aldH2454 comprised two new peaks (peaks 3 and 10 in Figure 3F) in the chromatogram, when compared to those of the control *E. coli* strain expressing crtM_SA_-crtN_SA_-crtP_SA_ (Figure 3A), as well as those from *E. coli* strains expressing *aldH420*, *aldH905*, or *aldH175*9 (Figure 3C–E). Further analysis using UV/VIS spectroscopy and LC/MS (Figure 3G,H) confirmed that peak 3 corresponded to 4,4-diaponeurosporenoic acid (**3**; [M + H]^−^ = 433.30) and peak 10 to 4,4′-diapolycopenoic acid (**10**; [M + H]^+^ = 431.30). Detection of 4,4′-diapolycopenoic acid (**10**) and 4,4′-diaponeurosporenoic acid (**3**) indicates that the AldH2454 enzyme reveals broad substrate specificity, similar to the CrtP2 enzyme, and functionally oxidizes 4,4′-diapolycopene-4-al (**7**) and 4,4′-diaponeurosporene-4-al (**2**) in *E. coli*. Although the carotenoid monoaldehyde 4,4′-diapolycopene-4-al (**7**) was not detected in the cell extract of both *E. coli* [pACM_crtM_SA_-crtN_SA_ + pUCM_crtP2_PF_] and [pACM_crtM_PF_-crtN_PF_-crtP2_PF_] (Figure 2), it is highly probable that AldH2454 acted on 4,4′-diapolycopene-4-al (**7**) before CrtP2 catalyzed the subsequent oxidation of 4,4′-diapolycopene-4-al (**7**) into 4,4′-diapolycopene-dial (**8**). Therefore, it is likely that AldH2454 was the second missing pathway enzyme involved in the oxidation reaction of 4,4′-diaponeurosporene-4-al (**2**) into 4,4′-diaponeurosporenoic acid (**3**) (Figure 1B), completing the biosynthesis of glycosyl-4,4′-diaponeurosporen-4′-ol-4-oic acid **(5)** in *P. faecalis*.

Eventually, a third enzyme may be required to catalyze the alcohol group introduced into one end of the 4,4′-diaponeurosporene backbone (a question mark in Figure 1B). Two possible routes are available for the formation of 4,4′-diaponeurosporen-4′-ol-4-oic acid (**4**): 1. an oxidative reaction [2], wherein an alcohol group is directly introduced into one end of 4,4′-diaponeurosporene (**1**), resulting in 4,4′-diaponeurosporen-4′-ol-4-oic acid (**4**), or 2. a reductive reaction of one aldehyde group [16] of either 4,4′-diaponeurosporene-dial or 4,4′-diaponeurosporen-4′-al-4-oic acid into 4,4′-diaponeurosporen-4′-ol-4-oic acid (**4**). Further research is underway to mine the corresponding enzymes.

## 3. Materials and Methods

### 3.1. Bacterial Strains Culture Condition and Plasmids

The bacterial strains and plasmids used in the present study are listed in Table 1. *E. coli* strain Top10 was used for gene cloning, and XL1-Blue was used for the expression of C30 carotenoid biosynthetic pathway genes. *E. coli* strains were aerobically cultured in Luria-Bertani (LB) medium at 30 °C on a rotary shaker at 250 rpm. Appropriate antibiotics ampicillin (100 μg/mL), chloramphenicol (50 μg/mL), and kanamycin (30 μg/mL) were supplemented as required. For carotenoid production, a preculture was grown in a 4 mL tube of Terrific Broth (TB) medium supplemented with 100 μg/mL ampicillin and/or 50 μg/mL chloramphenicol overnight at 30 °C by shaking at 250 rpm. Thereafter, the preculture was inoculated into a 300 mL baffle flask containing TB medium supplemented with the required antibiotics at 30 °C by shaking at 250 rpm for 36 h.

### 3.2. Genome Mining

A standalone basic local alignment and search tool program package (BLAST+) v2.2.31 (http://www.ncbi.nlm.nih.gov/) was locally installed and utilized to identify the missing pathway enzymes of *P. faecalis*. A local protein BLAST database of the *P. faecalis* genome (GenBank accession number CP019401) was generated by running the makeblastdb program [17]. Putative CrtP-like enzymes encoding 4,4-diaponeurosporene oxidase were explored by running the blastp program with default parameters against the local protein database, with the query amino acid sequence as that of CrtP (GenBank accession number ALY16520.1) from *Staphylococcus aureus*. Similarly, putative AldH-like enzymes were explored against the local protein database with the query amino acid sequence as that of the AldH enzyme (GenBank accession number BAF68130.1) from *S. aureus*.

### 3.3. Cloning and Construction of Expression Modules of Carotenoid Pathway Genes

Genomic DNA of *P. faecalis* was extracted using the Genomic DNA extraction kit (Macrogen, Seoul, South Korea). A *crtP2* gene encoding 4,4-diaponeurosporene oxidase (CrtP2) and four *aldH*-like genes encoding aldehyde dehydrogenase (AldH) were amplified from the genomic DNA using specific PCR primers (Table 2). Each PCR product was cloned into the corresponding sites of the constitutive expression vector pUCM [9], resulting in pUCM_X_y_ (where X is a cloned gene name and subscript Y is the bacterial source name) (Table 1)**.** In order to construct the 4,4′-diaponeurosporen-4′-al biosynthetic pathway, *crtP2* gene on pUCM_crtP2_PF_ was subcloned with the promoter and terminator sequences into pACM_crtM_PF_-crtN_PF_ [7], generating pACM_crtM_PF_-crtN_PF_-crtP2_PF_.

### 3.4. Isolation of Carotenoids

For carotenoid isolation, the cells and media were separated via centrifugation (4 °C, 4000 rpm). The pelleted cells were repeatedly extracted with 30 mL of acetone until all visible pigments were removed. Colored supernatants were pooled after centrifugation (4 °C and 3000 rpm) and concentrated into a small volume (1–2 mL) using a Genevac^TM^ EZ2-Plus centrifugal evaporator (Genevac, New York, NY, USA). Thereafter, 5 mL of ethyl acetate (EtOAc) was added to the concentrated solution and re-extracted after the addition of 5 mL NaCl (5 N) solution. Next, the upper organic phase containing carotenoids was collected, washed with distilled water, dehydrated by the addition of 0.5 g sodium sulfate, and completely dried using the EZ2-Plus evaporator. Dried samples were stored at −80 °C until further analysis.

### 3.5. Analysis of Carotenoids

A 10 μL aliquot of the carotenoid extracts was applied to a C18 reverse phase column, and then eluted under isocratic conditions with a solvent system (acetonitrile:methanol:2-propanol, 80:15:5) at a flow rate of 1 mL/min using an Agilent 1200 HPLC system (Agilent Technologies, Santa Clara, CA, USA) equipped with a photodiode array detector according to our previous paper [5]. The mass fragmentation spectra of carotenoids were monitored using both positive and negative ion modes in the mass range of 200–900 *m*/*z* on a liquid chromatography/mass spectrometry system (LC/MS; Agilent 6150, Agilent Technologies) equipped with an atmospheric pressure chemical ionization ion source according to our previous paper [8]. For structural elucidation, carotenoids were identified using a combination of HPLC retention times, UV/VIS absorption spectra, and mass fragmentation spectra.

## 4. Conclusions

In this study, the two missing genes, *crtP2* and *aldH2454*, in the glycosyl-4,4′-diaponeurosporen-4′-ol-4-oic acid biosynthetic pathway of *P. faecalis* were identified using a genome mining approach. CrtP2 and AldH enzymes were functional in heterologous *E. coli* and generated two carotenoid aldehydes (4,4′-diapolycopene-dial (**8**) and 4,4′-diaponeurosporene-4-al (**2**)) and two carotenoid carboxylic acids (4,4′-diaponeurosporenoic acid (**3**) and 4,4′-diapolycopenoic acid (**10**)). Both *crtP2* and *aldH2454* are remotely located from the carotenoid gene cluster of *P. faecalis*, similar to the C30 carotenoid pathway gene organization in *S. aureus* and *Methylomonas* sp.

## Figures and Tables

**Table 1:** Strains and plasmids used in this study.

Strains and Plasmids	Relevant Properties	Source or Reference
*E.coli* strains		
TOP10	F- mcrA Δ(mrr-hsdRMS-mcrBC) φ80lacZΔM15 ΔlacX74 recA1 araD139 Δ(ara-leu)7697 galU galK rpsL (Str^R^) endA1 nupG	Invitrogen
XL1-blue	endA1 gyrA96(nal^R^) thi-1 recA1 relA1 lac glnV44 F’[::Tn10 proAB^+^lacI^q^ (ΔlacZ)M15] hsdR17(r_K_^−^m_K_^+^)	Stratagene
Other bacteria strains		
*P. faecalis* AJ003^T^	Source for C_30_ carotenoid pathway genes	KCTC 32457
Plasmids		
pUCM	Cloning vector modified from pUC19. Constitutive *lac* promoter, Amp^R^	[9]
pACM	Expression vector modified form pACYC184; deleted *lacZ* fragment and lac promoter, Cm	[9]
pACM_crtM_SA_-crtN_SA_	Constitutively expressing *crtM* and *crtN* genes from *S. aureus*	[8]
pACM_crtM_SA_-crtN_SA_-crtP_SA_	Constitutively expressing *crtM*, *crtN* and *crtP* genes from *S. aureus*	[7]
pUCM_aldH_SA_	Constitutively expressing *aldH* gene from *S. aureus*	[8]
pUCM_crtP1_PF_	Constitutively expressing *crtP1* gene from *P. faecalis*	[7]
pUCM_crtP2_PF_	Constitutively expressing *crtP2* gene from *P. faecalis*	This study
pUCM_aldH420_PF_	Constitutively expressing *aldH420* gene from *P. faecalis*	This study
pUCM_aldH905_PF_	Constitutively expressing *aldH905* gene from *P. faecalis*	This study
pUCM_aldH1759_PF_	Constitutively expressing *aldH1759* gene from *P. faecalis*	This study
pUCM_aldH2454_PF_	Constitutively expressing *aldH2454* gene from *P. faecalis*	This study
pACM_crtM_PF_-crtN_PF_	Constitutively expressing *crtM* and *crtN* genes from *P. faecalis*	[7]
pACM_crtM_PF_-crtN_PF_-crtP2_PF_	Constitutively expressing *crtM*, *crtN* and *crtP* genes from *P. faecalis*	This study

**Table 2:** Primers used in this study.

Gene	Sequence (5′ to 3′) ^a^	Enzyme Site
*crtP2*	F: GCTCTAGAAGGAGGATTACAAAATGAATCATTCACAAAAATCG	XbaI
	R: CGGAATTCCTATTTCTTCTCTGCTTGAT	EcoRI
*aldH420*	F: GCTCTAGAAGGAGGATTACAAAATGCAACAGCATAAAATATATA	XbaI
	R: ATAAGAATGCGGCCGCTTATTTACTATTTTTATACTGCAT	NotI
*aldH905*	F: GCTCTAGAAGGAGGATTACAAAATGAAAAAACAGCAAATGTATG	XbaI
	R: ATAAGAATGCGGCCGCTTAATATTTGAGCGCTACATT	NotI
*aldH1759*	F: GCTCTAGAAGGAGGATTACAAATGAAAACCGATTTTTCAAAAAT	XbaI
	R: ATAAGAATGCGGCCGCTTATTTTTTGGTGTTAGTAACA	NotI
*aldH2454*	F: GCTCTAGAAGGAGGATTACAAAATGAATTTTACAGCAACTGAT	XbaI
	R: ATAAGAATGCGGCCGCTTATTTCAGTACTGTCTTGAT-3′	NotI

^a^ Underline indicates the site of restriction enzyme digestion.
